# Four-Period Vertically Stacked SiGe/Si Channel FinFET Fabrication and Its Electrical Characteristics

**DOI:** 10.3390/nano11071689

**Published:** 2021-06-28

**Authors:** Yongliang Li, Fei Zhao, Xiaohong Cheng, Haoyan Liu, Ying Zan, Junjie Li, Qingzhu Zhang, Zhenhua Wu, Jun Luo, Wenwu Wang

**Affiliations:** Integrated Circuit Advanced Process Center, Institute of Microelectronics, Chinese Academy of Sciences, Beijing 100029, China; liyongliang@ime.ac.cn (Y.L.); zhaofei@ime.ac.cn (F.Z.); liuhaoyan@ime.ac.cn (H.L.); zanying@ime.ac.cn (Y.Z.); lijunjie@ime.ac.cn (J.L.); zhangqingzhu@ime.ac.cn (Q.Z.); wuzhenhua@ime.ac.cn (Z.W.); luojun@ime.ac.cn (J.L.)

**Keywords:** stacked SiGe/Si, epitaxial grown, Fin etching, FinFET

## Abstract

In this paper, to solve the epitaxial thickness limit and the high interface trap density of SiGe channel Fin field effect transistor (FinFET), a four-period vertically stacked SiGe/Si channel FinFET is presented. A high crystal quality of four-period stacked SiGe/Si multilayer epitaxial grown with the thickness of each SiGe layer less than 10 nm is realized on a Si substrate without any structural defect impact by optimizing its epitaxial grown process. Meanwhile, the Ge atomic fraction of the SiGe layers is very uniform and its SiGe/Si interfaces are sharp. Then, a vertical profile of the stacked SiGe/Si Fin is achieved with HBr/O_2_/He plasma by optimizing its bias voltage and O_2_ flow. After the four-period vertically stacked SiGe/Si Fin structure is introduced, its FinFET device is successfully fabricated under the same fabrication process as the conventional SiGe FinFET. And it attains better drive current I_on_, subthreshold slope (SS) and I_on_/I_off_ ratio electrical performance compared with the conventional SiGe channel FinFET, whose Fin height of SiGe channel is almost equal to total thickness of SiGe in the four-period stacked SiGe/Si channel FinFET. This may be attributed to that the four-period stacked SiGe/Si Fin structure has larger effective channel width (W_eff_) and may maintain a better quality and surface interfacial performance during the whole fabrication process. Moreover, Si channel of the stacked SiGe/Si channel turning on first also may have contribution to its better electrical properties. This four-period vertically stacked SiGe/Si channel FinFET device has been demonstrated to be a practical candidate for the future technology nodes.

## 1. Introduction

High-mobility SiGe or Ge channel p-type Fin field effect transistor (FinFET) or gate-all-around (GAA) devices have been demonstrated to be a valid option as performance booster for future technology nodes [1,2,3]. The low-Ge-content SiGe channel will be implemented firstly on the FinFET owing to its advantages of higher hole mobility, better negative bias temperature instability (NBTI) reliability than silicon and more compatible with present silicon platform [4,5]. So far, a SiGe channel can be integrated in FinFET architectures in multiple ways, e.g., by shallow trench isolation (STI) last scheme [6] or by STI first strategy [7] or epitaxial cladding of Si Fins [8]. However, a high quality of low-Ge-content SiGe epitaxial grown on Si substrate is still a challenge task to solve the epitaxial thickness limit of SiGe film and the threading dislocations (TD) defects. This is because its theoretical critical thickness value is only ~10 nm [9]. Compared with the stable low-Ge-content SiGe layer, the thickness of metastable SiGe layer on Si can reach ~100 nm, but its quality is more easily affected by the following high temperature, implantation, and other processes. The other challenge about SiGe Fin channel is that it has relatively high interface trap charge (N_it_) at the interfacial layer (IL)/SiGe channel due to the undesired GeO_x_ formation [10]. The passivation techniques of SiGe layer, such as Si-cap, O_3_ low temperature oxidation, selective GeO_x_-Scavenging, and fluorine/nitrogen plasma treatment, have been studied and the experimental demonstration on low-N_it_ SiGe gate stack have been reported [11,12,13,14,15]. However, these passivation techniques may have compatibility problems with a state-of-the-art FinFET. Therefore, a high quality of low-Ge-content SiGe channel FinFET fabrication is still a challenge task and there are limited reports disclosing process details.

In this report, to solve the epitaxial thickness limit and the high interface trap density of SiGe channel FinFET, a four-period vertically stacked SiGe/Si multilayer with the thickness of each SiGe layer less than 10 nm grown on Si substrate is demonstrate by optimizing the epitaxial process. Then, an optimized stacked SiGe/Si Fin etching process with HBr/O_2_/He plasma is also introduced to attain a perfect profile. Finally, the four-period vertically stacked SiGe/Si channel FinFET is successfully fabricated and it achieves better drive current I_on_, subthreshold slope (SS) and I_on_/I_off_ ratio performance compared with the conventional SiGe channel FinFET under the similar fabrication process.

## 2. Materials and Methods

**Figure 1:**
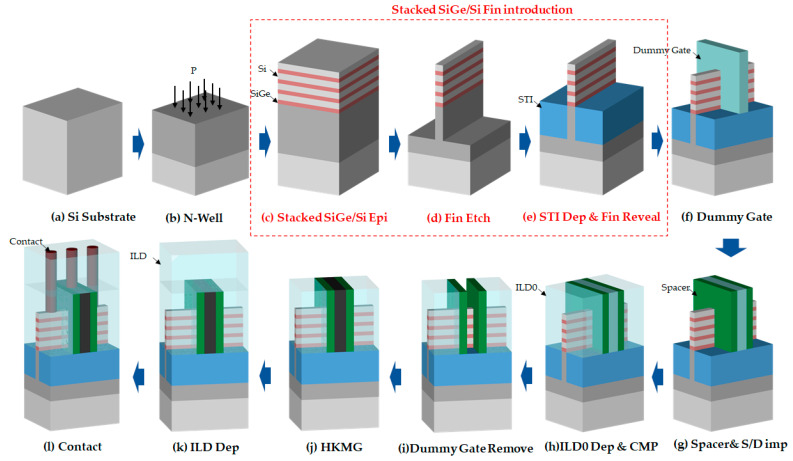
Fabrication flow of four-period stacked SiGe/Si Channel FinFET device.

P-type FinFET device with a four-period vertically stacked SiGe/Si channel was fabricated on 8-inch p-type (100) wafers. Its fabrication flow is shown in Figure 1, where the fundamental differences with the conventional SiGe channel FinFET process are the stacked SiGe/Si Fin introduction (indicated with red color).

After a standard nWell formation, four-period stacked SiGe/Si multilayer were epitaxially grown by reduced-pressure chemical vapor epitaxial deposition. Then, the vertical Fin pattern with stacked SiGe/Si multilayer on the top of Si substrate were formed by a spacer image transfer (SIT) technique under an optimal HBr/O_2_/He plasma. After STI filling and planarization, a low temperature of 850 °C for 30 s STI densification anneal and 1:100 diluted HF solution Fin reveal was implemented to attain a stacked SiGe/Si Fin formation [16]. Then, a low temperature SiO_2_ deposition and dummy gate patterning were performed. After spacer 1 and spacer 2 definition, lightly doped drain (LDD) and source and drain (S/D) implantation was implemented with B and BF_2_ dopant respectively. A low temperature dopant activation of 850 °C for 30 s was performed to keep the stacked SiGe/Si Fin stability [16]. Inter layer dielectric (ILD) deposition and CMP was employed to exposure the dummy gate. A standard tetramethylammonium hydroxide (TMAH) solution at 70 °C was used to remove the dummy poly gate with high selectivity to the underlying oxide on the stacked SiGe/Si Fin. After removal of this oxide, an in-situ low temperature O_3_ passivation treatment at 300 °C for 1 min was employed and the Al_2_O_3_/HfO_2_ high-k (HK) dielectric and TiN-based/W metal gate (MG) stack were deposited by the atomic-layer-deposition (ALD) tool. Finally, the standard FinFET following processing was employed to complete the stacked SiGe/Si channel FinFET device fabrication.

## 3. Result and Discussion

### 3.1. Epitaxial Growth of Stacked SiGe/Si Multilayer

After a standard HF-last clean, to maintain the well doping profile and attain an excellent surface of the Si substrate, a H_2_ pre-bake treatment of 825 °C for 5 min is performed. The epitaxial growth of stacked SiGe/Si multilayer is prepared using dichlorosilane (DCS) and GeH_4_ as SiGe layer precursors and SiH_4_ as Si layer precursor at 650 °C in H_2_ ambient, respectively. And a four-period stacked SiGe/Si is fabricated successfully on the Si substrate by appropriately exchanging the sequences of introduced gases.

**Figure 2:**
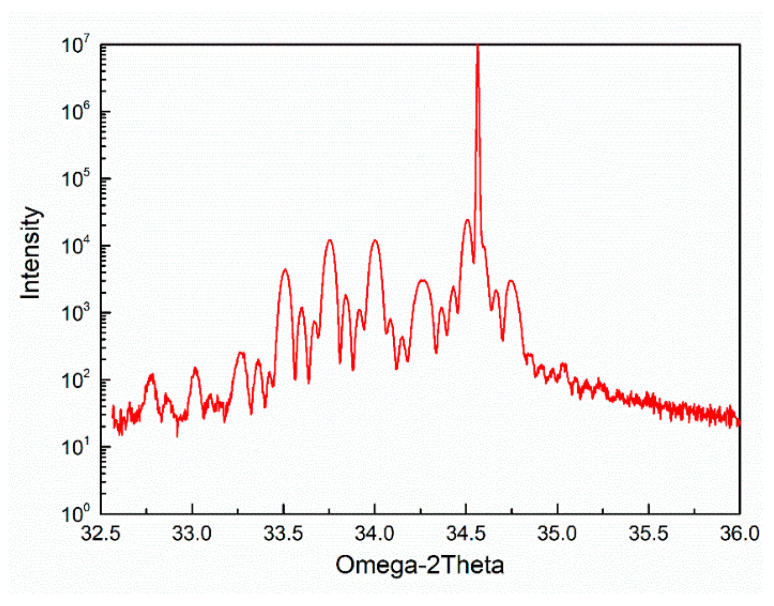
HRXRD spectra result on the four-period stacked SiGe/Si multilayer epitaxial grown on Si substrate.

The crystalline microstructure of the four-period stacked SiGe/Si multilayer epitaxial grown on Si substrate is detected by high resolution X-ray diffraction (HRXRD, Bruker, Tel Aviv, Israel) in the vicinity of the (004) Bragg peak with a Cu peak radiation. Its ω − 2θ HRXRD scan result is shown in Figure 2. It is worth to note that a series of obvious high-intensity satellite peaks are found, indicating that the epitaxial layers of the four-period stacked SiGe/Si multilayer are under strained due to the lattice constant mismatch of Si and SiGe. Moreover, the presence of small-intensity thickness fringes is the characteristic of high quality of the stacked SiGe/Si multilayer.

**Figure 3:**
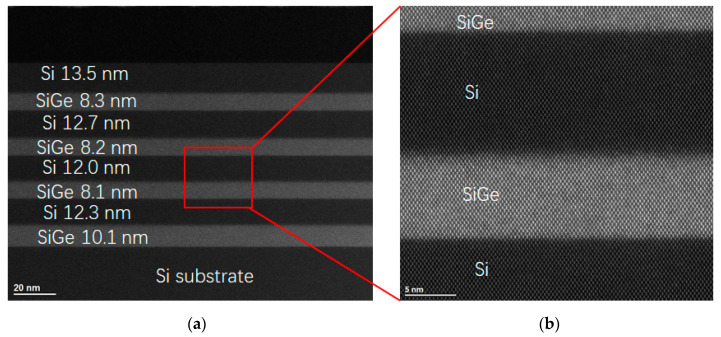
(**a**)Cross-section HAADF-STEM images of four-period stacked SiGe/Si multilayer; (**b**) its magnified images at the SiGe/Si interfaces.

In addition, its high-angle annular dark field scanning transmission electron microscopy (HAADF-STEM, FEI Talos, Brno, Czech Republic) analysis results are shown in Figure 3. It is found that there are no misfits at the SiGe/Si interfaces, nor threading dislocations crossing the stacked SiGe/Si epitaxial film. Therefore, a high crystal quality four-period stacked SiGe/Si multilayer with thin and distinct interfaces between SiGe and Si is successfully prepared. Meanwhile, the thickness of SiGe from top to bottom is 8.3, 8.2, 8.1 and 10.1 nm under the same time of epitaxial grown. Namely, the thickness of bottom SiGe is ~2 nm thicker than that of others. It is known that the epitaxial rate is strongly dependent on the crystallization of under-layer, that is, the epitaxial rate might be decreased if multi-crystallization occurs in the under-layer.

**Figure 4:**
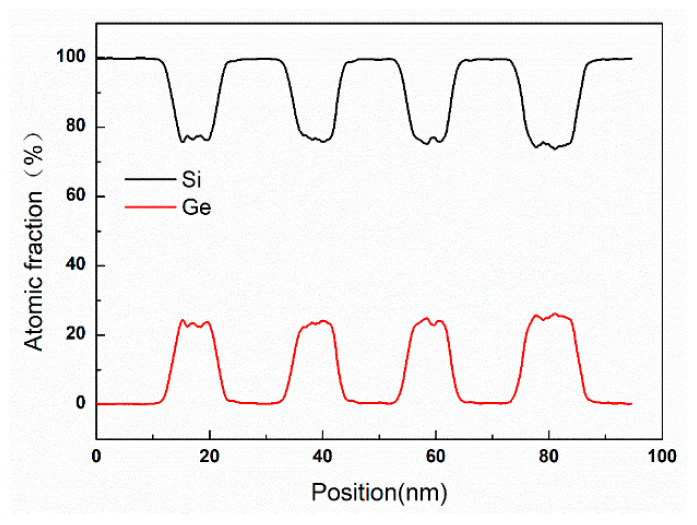
EDX line scan analysis of Ge and Si elements across the four-period stacked SiGe/Si multilayer.

Subsequently, EDX (FEI Talos, Brno, Czech Republic) line scan analysis of Ge and Si elements is also performed to determine the interfacial morphologies, and atomic fraction of the SiGe/Si layers for the four-period stacked SiGe/Si multilayer. The result is shown in Figure 4. It can be observed that the Ge atomic fraction of the SiGe layers from top to bottom are 23.2%, 23.5%, 23.8%, and 24.5%, respectively. Moreover, the width of transition layer of Si and SiGe are within 1.5 nm. Therefore, very uniform Ge atomic fraction of the SiGe layers with sharp SiGe/Si interfaces are achieved.

### 3.2. Stacked SiGe/Si Fin Etching

**Figure 5:**
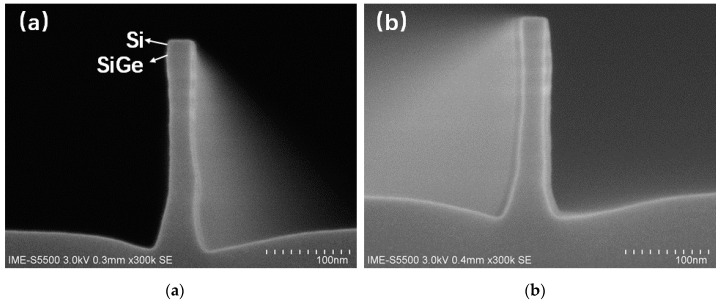
Scanning electron microscope (SEM) images of Fin profile under HBr/O_2_/He plasma under (**a**) bias voltage of −70 V and O_2_ flow of 2.2 sccm, (**b**) bias voltage of −90 V and O_2_ flow of 2.5 sccm.

Based on previous SiGe Fin etching result, HBr-based plasma is chosen as the etching gas for the four-period stacked SiGe/Si multilayer. Figure 5 presents the profiles of Si substrate/four-period stacked SiGe/Si multilayer Fin produced by HBr/O_2_/He plasma under different bias voltage and O_2_ flow. The other etching process conditions are as follows: top power of 350 W, pressure of 6 mTorr, HBr flow of 110 sccm, He flow of 50 sccm. A more vertical profile of the Si substrate/four-period stacked SiGe/Si multilayer Fin structure can be achieved as its bias voltage increase from −70 V to −90 V and its O_2_ flow increasing from 2.2 to 2.5 sccm. This is because that increasing the bias voltage can attain a larger ion bombardment effect and slightly increasing O_2_ flow can help promote passivation films formation on the stacked SiGe/Si Fin sidewall and preserve its profile during etching.

### 3.3. FinFET Device Fabrication

**Figure 6:**
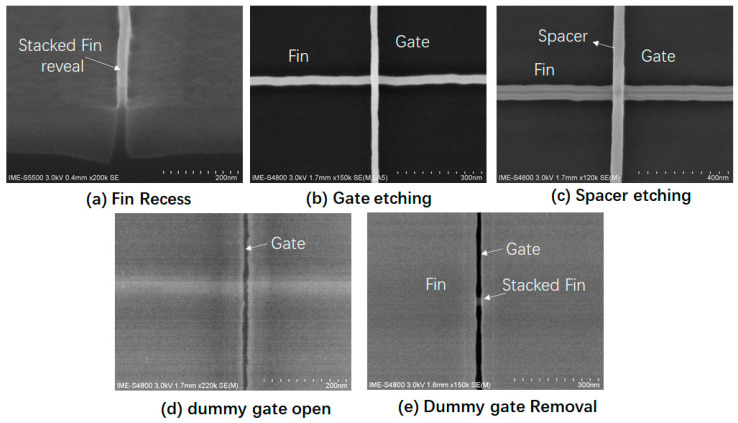
SEM images of stacked SiGe/Si channel FinFET device at different fabrication stages: (**a**) Fin reveal post STI recess, (**b**) dummy gate formation, (**c**) spacer formation, (**d**) poly gate open by CMP, (**e**) dummy gate removal.

**Figure 7:**
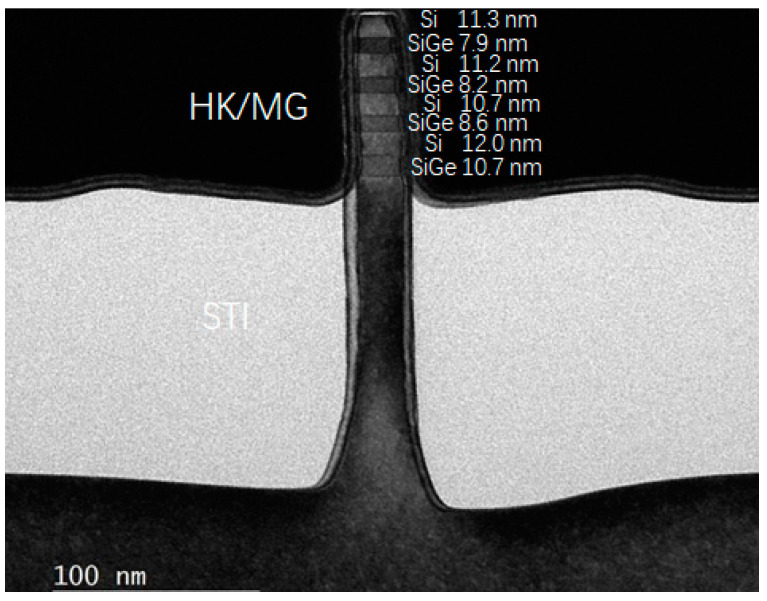
TEM result of four-period stacked SiGe/Si channel FinFET under the HK/MG stack at the end of fabrication processing.

**Figure 8:**
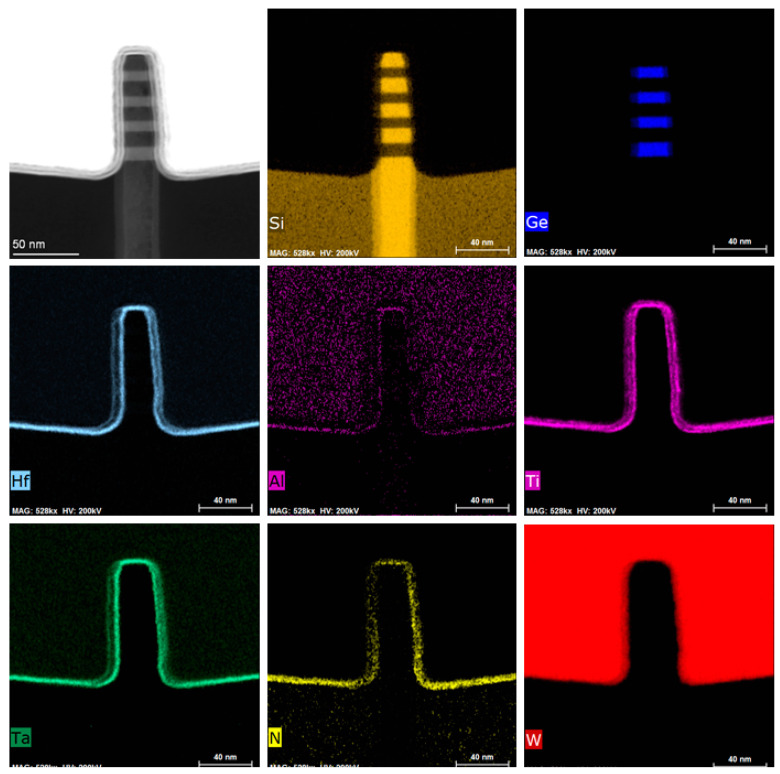
HAADF-STEM and EDS mapping results of the four-period stacked SiGe/Si channel FinFET under the HK/MG stack at the end of processing.

After these above newly developed epitaxial growth and etching technologies are implemented, the results of following major fabrication process of the four-period stacked SiGe/Si channel FinFET device are shown in Figure 6. Figure 6a presents the cross-sectional SEM image of the Fin reveal structure post STI recess by 1:100 diluted HF solution. It is found that the STI is well controlled and the stacked SiGe/Si Fin on the top of the Si has been revealed. Figure 6b shows the top view SEM image of stacked SiGe/Si channel FinFET device after gate formation with critical dimension (CD) of ~25 nm. And conformal spacer 1 and spacer 2 in Figure 6c are realized at two sides of dummy gate. Figure 6d,e present a top view of stacked SiGe/Si channel FinFET device after dummy gate CMP and dummy gate removal. As can be seen from the images, the surface of ILD is very smooth, and a dummy poly gate is successfully removed with a stacked SiGe/Si channel exposure in the open gate trench. After dummy gate removal, an in-situ low temperature O_3_ passivation treatment at 300 °C for 1 min and the Al_2_O_3_/HfO_2_ and TiN/TaN/TiN//W are implemented as HK and MG, respectively. Figure 7 shows cross-sectional transmission electron microscopy (TEM) image for the four-period stacked SiGe/Si channel under the HK/MG stack at the end of fabrication processing. And its HAADF-STEM and EDS mapping results are shown in Figure 8. It is found that a perfect four-period stacked SiGe/Si channel Fin structure with stable SiGe and Si layers is realized and the multilayer HK/MG are well wrapped around. At the same time, the Fin height of SiGe channel is 80.6 nm and the CD is 20 nm.

### 3.4. Electrical Performance

As a comparison, the conventional SiGe channel FinFET is also fabricated under the similar fabrication process.

**Figure 9:**
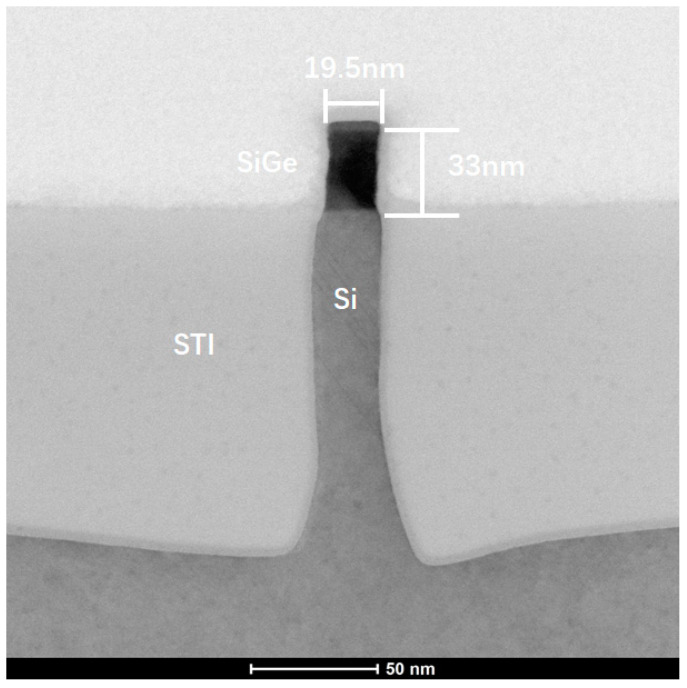
TEM result of the conventional SiGe channel Fin profile.

As shown in Figure 9, the Fin height of its SiGe channel is 33 nm and the CD is 19.5 nm. The Fin height of SiGe channel is almost equal to total thickness of SiGe in the four-period stacked SiGe/Si channel FinFET. Moreover, the CD and profile of Fin of these two kinds of FinFET device are almost comparable. Therefore, these two kinds of FinFET device have almost the same footprint.

**Figure 10:**
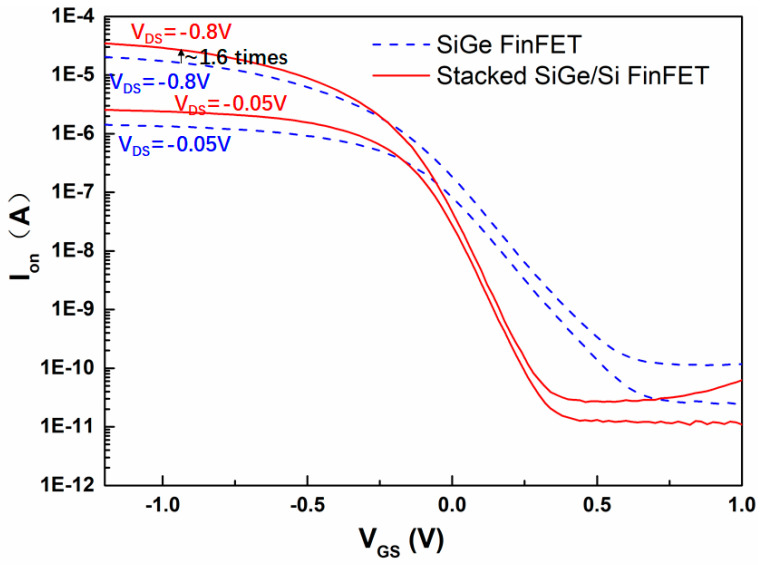
I_DS_-V_GS_ characteristics of the four-period stacked SiGe/Si channel FinFET and conventional SiGe channel FinFET under the similar fabrication process.

Figure 10 shows the I_DS_-V_GS_ curves for the four-period stacked SiGe/Si channel FinFET and conventional SiGe channel FinFET. Compared with conventional SiGe channel FinFET, the I_on_, SS and I_on_/I_off_ ratio of the four-period stacked SiGe/Si channel FinFET under the same footprint show obvious benefit. And their electrical characteristic data are summarized in Table 1. Its I_on_ under V_DS_ = V_GS_ = −0.8 V increases 1.6 times, improved from 13.3 µA to 21.2 µA, and its I_on_/I_off_ ratio can be improved from 1 × 10^5^ to 1 × 10^6^ due to the increase of I_on_ and the decrease of I_off_ at the same time. At the same time, the threshold voltage (V_tsat_) extracting at I_on_ of 1 × 10^−9^ A can be improved from +0.38 V to +0.16 V. In addition, four-period stacked SiGe/Si channel FinFET is easier to obtain a better SS characteristic than the conventional SiGe FinFET under the same unoptimized O_3_ passivation process. Its SS can be improved from 149 mV/dec to 90 mV/dec. The better SS characteristic should be related to the four-period stacked SiGe/Si Fin structure and it also can help to increase the I_on_ and decrease the I_off_. The better SS can be attributed to the following two reasons: first, the stacked SiGe/Si with each layer SiGe of 8–10 nm in the stable stage may maintain a better quality and surface interfacial performance during the whole fabrication process compared with the the conventional SiGe of 33 nm in the metastable stage; second, the Si channel of the stacked SiGe/Si channel may turn on first due to its lower D_it_. Moreover, the larger Ion can be attributed to the increasing of effective channel width (W_eff_) because the W_eff_ of four-period stacked SiGe/Si Fin is 181 nm and the W_eff_ of conventional SiGe Fin is only 85.5 nm.

This four-period stacked SiGe/Si channel FinFET has been demonstrated to be a practical candidate for the future technology nodes. However, it is important to emphasize that these results are preliminary for the four-period stacked SiGe/Si channel FinFET, and there is still much room to improve its electrical characteristic, such as its SS and V_tsat_. Therefore, we will employ the interfacial passivation, and gate stack engineering to further optimize its electrical performance in the future.

## 4. Conclusions

In a summary, a four-period vertically stacked SiGe/Si FinFET device was successfully fabricated by optimizing its epitaxial grown and Fin etching process. Compared with the conventional SiGe channel FinFET under the same footprint, its I_on_ increases 1.6 times, I_on_/I_off_ ratio increases 1 order and SS can be improved from 149 to 90 mV/dec because the four-period stacked SiGe/Si Fin structure has larger W_eff_ and may maintain a better quality and surface interfacial performance during the whole fabrication process. Therefore, this device has been demonstrated to be a practical candidate for future technology nodes. Moreover, considering the compatibility of fabrication process, it also can be use as the I/O device for the vertically stacked Gate-All-Around horizontal nanowire/sheet technology if a thicker gate stack is employed and the channel release step is skipped.

## Figures and Tables

**Table 1:** Electrical characteristic comparison of the four-period stacked SiGe/Si and conventional SiGe channel FinFET.

Category	Ion (µA)	SS (mV/dec)	V_tsat_ (V)	I_on_/I_off_
Conventional SiGe channel FinFET	13.3	149	0.38	1 × 10^5^
Four-period stacked SiGe/Si channel FinFET	21.2	90	0.16	1 × 10^6^

## Data Availability

Not applicable.
